# Dissociations within neglect-related reading impairments: Egocentric and allocentric neglect dyslexia

**DOI:** 10.1080/13803395.2020.1715926

**Published:** 2020-02-17

**Authors:** Margaret Jane Moore, Nir Shalev, Celine R. Gillebert, Nele Demeyere

**Affiliations:** a Department of Experimental Psychology, Radcliffe Observatory Quarter, University of Oxford, Oxford, UK; b Department of Brain and Cognition, KU Leuven, Leuven, Belgium

**Keywords:** Neglect dyslexia, visuospatial neglect, acquired dyslexia, sentence-level neglect

## Abstract

Consistently lateralized reading errors are commonly understood as side-effects of visuospatial neglect impairment. There is however a qualitative difference between systematically omitting full words presented on one side of passages (egocentric neglect dyslexia) and lateralized errors when reading single words (allocentric neglect dyslexia). This study aims to investigate the relationship between egocentric and allocentric neglect dyslexia and visuospatial neglect.

1209 stroke survivors completed standardized reading and cancellation tests. Stringent criteria identified unambiguous cases of allocentric neglect dyslexia (N = 17) and egocentric neglect dyslexia (N = 35). These conditions were found to be doubly dissociated with all cases of egocentric and allocentric neglect dyslexia occurring independently. Allocentric neglect dyslexia was dissociated from both egocentric and allocentric visuospatial neglect. Additionally, two cases of allocentric neglect dyslexia which co-occurred with oppositely lateralized domain-general visuospatial neglect were identified. Conversely, all cases of egocentric neglect dyslexia were found in the presence of domain-general visuospatial neglect. These findings suggest that allocentric neglect dyslexia cannot be fully understood as a consequence of visuospatial neglect. In contrast, we found no evidence for a dissociation between egocentric neglect dyslexia and visuospatial neglect. These findings highlight the need for new, neglect dyslexia specific rehabilitation strategies to be designed and tested.

## Introduction

**Figure 1.**
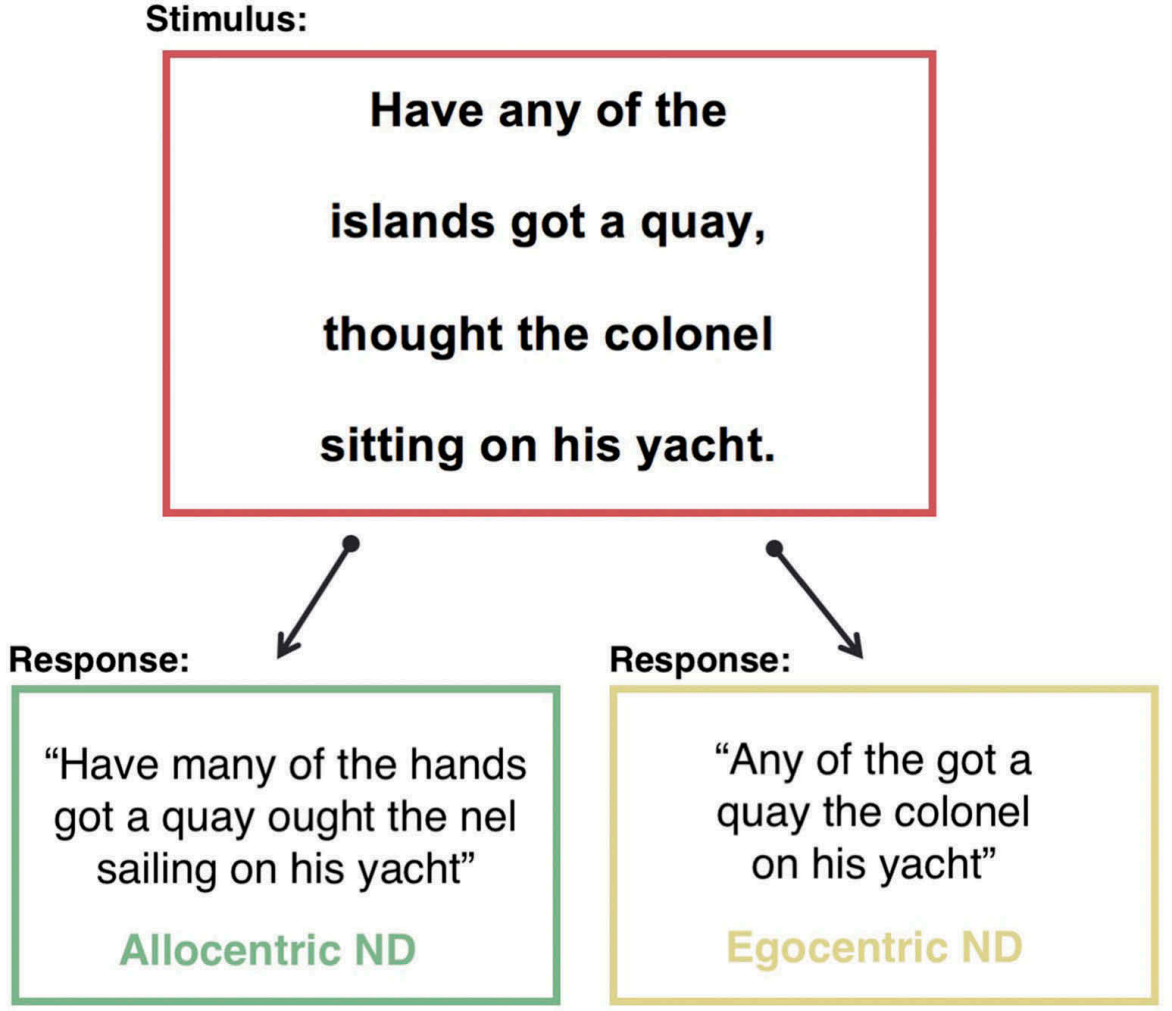
An illustration of the differential reading error pattern observed in patients with neglect dyslexia and sentence-level neglect. ND = Neglect Dyslexia.

Visuospatial neglect is a common neuropsychological syndrome characterized by a failure to attend to stimuli presented on one side of space (Halligan, Fink, Marshall, & Vallar, 2003; Parton, Malhotra, & Husain, 2004). This spatial-attentional deficit has been associated with a wide range of difficulties in activities of daily life in stroke survivors, including problems with reading books, menus, road signs, or other spatially presented written stimuli (Beschin et al., 2014; Ellis & Young, 2013; Turton et al., 2009). Previous neuropsychological research has identified two main categories of reading impairment behavioral phenotypes related to visuospatial neglect: egocentric (body-centered) and allocentric (object-centered) (Beschin et al., 2014; Ellis, Flude, & Young, 1987; Vallar, Burani, & Arduino, 2010; Young, Newcombe, & Ellis, 1991). Allocentric neglect dyslexia is characterized by consistently lateralized omission, addition, and substitution errors when reading individual words (Ellis et al., 1987; Hillis & Caramazza, 1995; Vallar et al., 2010). Conversely, patients with egocentric neglect dyslexia commit lateralized full word omissions when reading spatially presented passages of prose (Beschin et al., 2014) (see Figure 1 for an illustration of error patterns). Both egocentric and allocentric neglect dyslexia can impact either left- or right-lateralized stimuli (Vallar et al., 2010).

Egocentric and allocentric neglect dyslexia can co-occur within the same patient (for a review see Moore & Demeyere, 2017). For exampleYoung et al. (1991) identified a patient (VB) who committed both right-lateralized letter omission errors when reading single words and full-word omission errors on the right side of lines of prose. Despite these observed associations in some patients, other studies have found dissociations, suggesting that egocentric and allocentric neglect dyslexia represent independent cognitive impairments. Several case studies have identified patients exhibiting an allocentric neglect dyslexia reading impairment in the absence of egocentric reading impairment (e.g., Friedmann & Nachman-Katz, 2004; Moore & Demeyere, 2018). Friedmann and Nachman-Katz (2004) identified an allocentric neglect dyslexic patient (NT) who consistently omitted the left portion of words throughout spatially presented lines of prose, but never committed full-word omission errors. A behavioral dissociation between egocentric and allocentric neglect impairment has also been identified in group studies, notably by Beschin et al. (2014) who assessed 30 patients exhibiting left-lateralized egocentric (body-centered) visuospatial neglect deficits on a series of single word and passage reading tasks. Of these patients, 17 committed both egocentric and allocentric reading errors while 5 patients exhibited egocentric neglect dyslexia in the absence of allocentric-level impairment and 4 patients presented with allocentric neglect dyslexia in the absence of egocentric impairment. These findings illustrate that despite an apparent core egocentric visuospatial attentional deficit, egocentric and allocentric neglect dyslexia can result from spatial-attentional biases in different reference frames, supporting the conclusion that these conditions must represent independent impairments. However, it remains unclear whether these impairments are best characterized as separable consequences of a domain general visuospatial impairment or whether allocentric neglect dyslexia impairment is driven by a content-specific (word-specific) deficit.

Neglect-related reading impairments are frequently understood as side effects of visuospatial neglect rather than content-specific cognitive problems (Ellis & Young, 2013; Jackson & Coltheart, 2013; Mozer & Behrmann, 1990; Riddoch, 1990; Shallice, 1988). According to this logic, visuospatial neglect impairs the early visual processing of written stimuli’s perceptual features, leading to consistently lateralized reading errors (Jackson & Coltheart, 2013; Riddoch, 1990; Shallice, 1988). This argument is supported by findings that a neglect-related reading impairment commonly co-occurs with domain-general visuospatial neglect (Lee et al., 2009; Vallar et al., 2010) and that the severity of egocentric neglect acts as a significant predictor of allocentric neglect dyslexic reading impairment (Beschin et al., 2014; Lee et al., 2009). Lee et al. (2009) assessed a cohort of patients on standardized neglect tests and a single-word reading task finding that patients exhibiting allocentric neglect dyslexia impairment had significantly more severe egocentric neglect than patients who did not make neglect dyslexia reading errors. However, both Lee et al. (2009) and Beschin et al (2014), did not include tests for domain-general allocentric (object-centered) neglect, meaning that the relationship between allocentric neglect dyslexia and allocentric neglect remains unclear. Ptak, Di Pietro, and Schnider (2012) investigated reading impairments in a cohort of egocentric neglect patients and found that while the prevalence of full-word omissions (egocentric neglect dyslexia) was mediated by the egocentric location of stimuli, the incidence of allocentric neglect dyslexia word reading errors was not significantly affected by where stimuli were presented in space (Ptak et al., 2012). This strongly suggests that while egocentric neglect dyslexia may be explained as a side-effect of an egocentric neglect impairment, allocentric neglect dyslexia is likely caused by a differential spatial-attentional bias. This conclusion is further supported by the findings of meta-analyses which have revealed that while allocentric neglect dyslexia does frequently co-occur with egocentric visuospatial neglect, many cases of allocentric neglect dyslexia occur in the absence of this domain-general impairment (Moore & Demeyere, 2017, 2018; Vallar et al., 2010).

However, visuospatial neglect represents a highly heterogeneous condition with some patients exhibiting spatial-attentional biases within an egocentric reference frame and others within an allocentric frame of reference (Baylis, Baylis, & Gore, 2004; Bickerton, Samson, Williamson, & Humphreys, 2011; Demeyere & Gillebert, 2017; Hillis & Caramazza, 1995; Moore, Vancleef, Shalev, Husain, & Demeyere, 2019). Patients with allocentric neglect fail to notice portions of individual objects, regardless of where objects are presented in egocentric space (Demeyere & Gillebert, 2019). This object-centered neglect impairment can potentially provide a plausible explanation for some cases of allocentric neglect dyslexia. For example, patient SP (Young et al., 1991) was found to commit similar frequencies of left allocentric neglect dyslexia errors when reading in both the left and right visual field, suggesting that the spatial bias seen in allocentric neglect dyslexia can operate independently of egocentric spatial coordinates. This error pattern can be best understood as an impairment occurring within an object-centered reference frame (Haywood & Coltheart, 2000; Hillis & Caramazza, 1995), or within a smaller attentional window (Driver & Pouget, 2000). Unfortunately, SP did not complete neglect assessments which were sensitive enough to detect and differentiate between allocentric neglect and egocentric neglect, meaning that the relationship between allocentric neglect and neglect dyslexia remains unclear.

Indeed, the vast majority of existing allocentric neglect dyslexia case studies have not included object-centered neglect assessments (Moore & Demeyere, 2017; Vallar et al., 2010), meaning that the relationship between allocentric neglect and neglect dyslexia reading errors is not yet well understood. However, single case of allocentric neglect dyslexia occurring in the absence of ego- and allocentric neglect was recently documented by Moore and Demeyere (2018), providing strong evidence for a content-specific deficit. Particularly both patient AB (Moore & Demeyere, 2018) and patient NG (Caramazza & Hillis, 1990) demonstrated a consistent deficit with the terminal letters of words, regardless of orientation (vertical writing, and mirror reflected writing), in line with a word-specific deficit. The question remains whether this dissociation can be identified in a substantial portion of allocentric neglect dyslexia patients.

The purpose of the present study is to investigate the relationship between domain-general visuospatial neglect and neglect-related reading errors. Specifically, we aimed to elucidate the relationship between egocentric and allocentric and domain-general neglect to determine whether neglect dyslexia reading errors can be adequately accounted for as a consequence of domain-general neglect impairment or whether these reading errors are best understood as an independent, content-specific impairment.

## Methods

### Methods/materials

This study considers data collected as a component of two large cognitive screening cohort studies which conducted the Birmingham Cognitive Screen (BCoS; Humphreys, Bickerton, Samson, & Riddoch, 2012) or the Oxford Cognitive Screen (OCS) (Demeyere, Riddoch, Slavkova, Bickerton, & Humphreys, 2015). Both the OCS and BCoS are clinical neuropsychological screening tools. Though the BCoS is longer and has more subtests than the OCS, both screening tools provide a multi-domain summary of cognitive impairments following brain injury. Data from these assessments’ Reading, Cancellation, and Visual Field tasks was used in this experiment.

**Figure 2.**
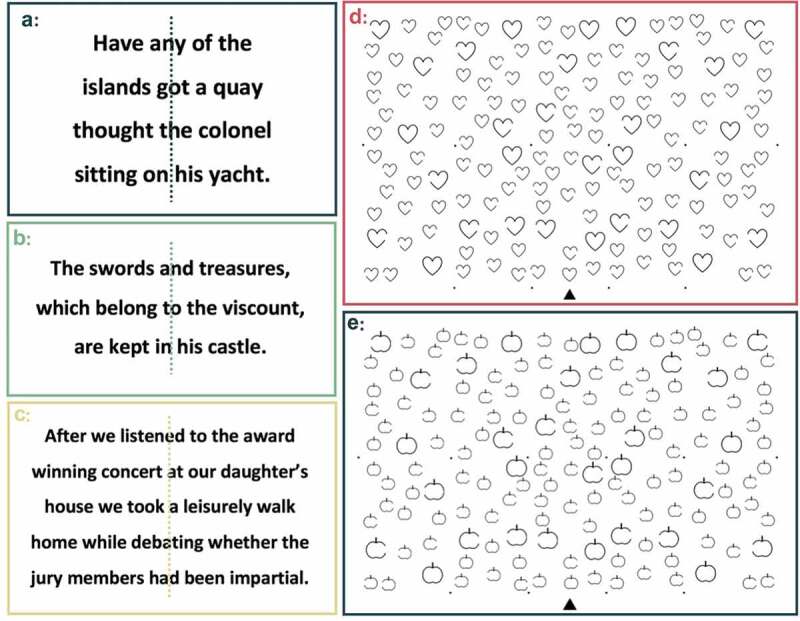
OCS and BCoS Reading Tasks and Cancellation Task stimuli. (a) and (d) are OCS stimuli while (b,c) and (e) are taken from the BCoS. Cancellation Task scoring gridline locations are denoted by the black dots within the search matrix. Sentence midpoints are denoted by dotted lines. These lines are not present during task administration, but were used to score patient performance. According to BCoS scoring guidelines, one point is assigned for correctly reading “daughter” and another for this word’s corresponding possessive suffix.

The BCoS Reading Task consists of one 14- and one 28-word sentence. The OCS Reading Task consists of one 15-word sentence. Each of these sentences is printed in size 26 font in the center of a full A4 sheet of paper (Figure 2). OCS and BCoS both contain a neglect cancellation task to measure both ego- and allocentric neglect, in a similar way to the gap detection task proposed by Ota, Fujii, Suzuki, Fukatsu, and Yamadori (2001). In these cancellation tasks, patients are presented with a search matrix including 150 line drawings of simple objects pseudorandomly distributed across a full A4 page presented in landscape orientation (Figure 2). One third of these drawings have left-lateralized gaps, one third have right gaps, and the remaining third are complete drawings. In both tasks, patients are asked to cross out all complete drawings while ignoring the incomplete, distractor stimuli. These drawings are arranged in a grid pattern to ensure that the probability for omissions in each section of the matrix is equal. This grid is not visible when completing the task, but is used to assign quantitative neglect asymmetry scores. Patients are shown examples of distractor and target drawings and given two practice trials before proceeding to the full task. The BCoS and OCS Cancellation Tasks have been shown to be highly sensitive and are to reliably differentiate between allocentric and egocentric visuospatial neglect deficits (Bickerton et al., 2011; Demeyere & Gillebert, 2019).

In the Reading Tasks, each patient was asked to read the presented sentences aloud as an examiner recorded their responses. Patients who misread or failed to read more than one word on the BCoS or OCS Reading Tasks were considered to be significantly impaired following the subtests’ published cutoffs. For patients with significant reading impairment, the severity of this impairment was scored as each patient’s total percent of words read correctly to facilitate comparisons across the two reading tests. For the cancellation tasks, patients were allowed three minutes to complete, patients who were unable to hold a pen responded by pointing to each stimulus which was then marked by the examiner. Egocentric neglect impairment was scored by subtracting the number of correctly identified targets on the left side of the page (max 20) from those correctly identified on the right side of the page (max 20, see Figure 2(d,e)). Allocentric neglect impairment was calculated by subtracting the number of right-gap false positive responses from the number of left-gap false positives. Egocentric asymmetry of less than −3 or greater than 3 or allocentric asymmetry of less than −1 or greater than 1 were considered to represent neglect impairment, following the published cut off values (Demeyere et al., 2015). Negative asymmetry scores denote right-lateralized neglect deficits and positive asymmetry scores denote left-sided neglect.

Patients also completed a short Visual Field test as a component of these standardized batteries. In this task, the examiner sat approximately one meter in front of the patient, asked the participant to fixate on the examiner’s nose, and sequentially wiggled their fingers in each of the patient’s visual quadrants. Failure to report one or more of these confrontations indicated significant visual field impairment.

### Neglect-related reading error identification

Error patterns on the reading tasks were systematically analyzed to identify clear cases of egocentric and allocentric neglect dyslexia. There are no existing standardized scoring procedures for diagnosing neglect-related reading impairments in stroke survivors. Therefore, this investigation employed a combination of rigorous quantitative and qualitative error evaluation procedures to identify patients with egocentric anc allocentric neglect dyselxia from these brief subtests.

**Figure 3.**
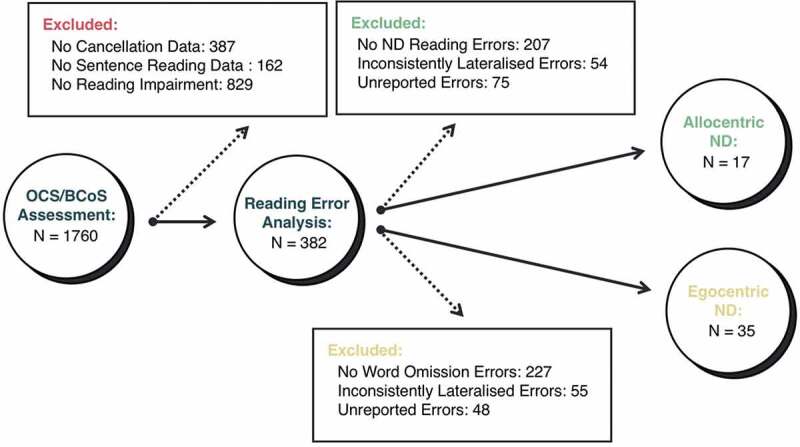
A visualization of the neglect dyslexia and sentence-level neglect patient identification process and patient exclusion counts at each stage. ND = Neglect Dyslexia.

A multi-step filtering process (see Figure 3) was followed to identify patients who demonstrated neglect-related reading impairments. First, only patients with a significant reading impairment according to OCS scoring guidelines were included. Second, only patients who committed more than one reading error which was clearly characteristic of egocentric or allocentric neglect dyslexia impairment were included. For allocentric neglect dyslexia, patients were only included if they committed two separate word reading errors demonstrating lateralized letter omission, addition, or substitution errors. For egocentric neglect dyslexia, patients were only included if they committed at least two full-word omission errors. As the reading data used in this investigation represents existing data from clinical cognitive assessments, some reading errors were scored binarily (correct/incorrect) with no qualitative error information was provided. Patients with no available qualitative information about the precise reading errors made were excluded from this investigation at this stage.

Third, potential egocentric and allocentric neglect dyslexia patients were only included if their error patterns were consistently lateralized. This was defined as demonstrating the same lateralization in at least 80% of potential egocentric or allocentric neglect dyslexia reading errors Finally, all potential neglect dyslexia reading patterns were qualitatively and independently reviewed by all 4 authors and agreed by consensus before including each patient in subsequent analyses. See Figure 3 for a visualization of this selection process and for patient exclusion counts at each stage.

We note that this represents a very conservative approach, aimed at only including those patients with very clear impairment. Similarly, this procedure is less likely to identify cases of co-occurring egocentric and allocentric neglect dyslexia than it is to identify either deficit in isolation. This is because in egocentric neglect dyslexia patients will, by definition, only read a portion of the presented words and patients with allocentric neglect dyslexia generally only commit neglect dyslexia errors in a portion of read words (Caramazza & Hillis, 1990; Vallar et al., 2010). Since at least two potential allocentric neglect dyslexia errors must be committed to confidently identify this reading impairment, the probability of detecting a patient exhibiting both impairments simultaneously on the OCS/BCoS reading tasks is low. However, previous research has established that egocentric and allocentric neglect dyselxia do commonly co-occur (Beschin et al., 2014; Ellis et al., 1987; Moore & Demeyere, 2017; Vallar et al., 2010). This investigation therefore explicitly aims to identify cases in which these conditions do NOT co-occur and does not attempt to make any claims about the prevalence of neglect dyslexia or what proportion of egocentric and allocentric neglect dyselxia occur independently.

### Participants

The BCoS was administered to 912 subacute stroke survivors (<3 months post-stroke) at different hospital settings across the West-Midlands, as part of the BUCS study (REC reference 08/H0301/6), coordinated by the University of Birmingham between 2006 and 2011. The OCS was administered to a consecutive sample of 848 stroke survivors (<3 weeks post-stroke) following admission to Oxford’s John Radcliffe Hospital acute stroke ward between 2012 and November 2017 as part of the Oxford Cognitive Screening programme (REC reference 11/WM/0299 and REC reference 14/LO/0648). Each participant provided verbal or written consent before beginning these cognitive assessment batteries. This resulting sample of 52 patients had an average age of 68.6 (Range = 18–92) and contained 34 (65.4%) women. See Table 1 for a breakdown of demographics and stroke types for each patient group.

**Table 1.** Demographics for patients with neglect dyslexia and sentence-level neglect. Unreported patient information represents missing demographics which were not recorded in medical notes. R = Right, L = Left, B = Bilateral, M = Male, F = Female, Isc = Ischemic, H = Hemorrhage, TIA = Transient Ischemic Attack.

Patient Group			
	Neglect Dyslexia	Sentence- Level	Total
**N:**	17	35	52
**Age:**			
mean (std)	70.2 (18.4)	67.9 (13.4)	68.6 (15.1)
**Gender:**			
	14F/3M	20F/15M	34 F/18 M
**Hand:**			
	15 R/0L/0B	27R/2L/1B	42R/2L/1B
**Education:**			
mean (std)	10.8 (2.08)	12.4 (3.93)	11.6 (3.52)
**Stroke Side:**			
	5R/8L/0B	27R/1L/4B	32R/9L/4B
**Stroke Type:**			
	9 Isc/2H/3 TIA	26 Isc/3H/2 TIA	35 Isc/5H/5 TIA

## Results

In this sample, 17 patients with allocentric neglect dyslexia (14 right and 3 left) and 35 cases of egocentric neglect dyslexia (1 right and 34 left) were identified according to this investigation’s rigorous inclusion criteria. Egocentric and allocentric neglect dyslexia were found to be behaviorally doubly dissociated with all cases of allocentric neglect dyslexia occurring independently of egocentric neglect dyslexia. Egocentric neglect dyslexia was not found to be dissociable from domain-general visuospatial neglect impairment. All 35 egocentric neglect dyselxia patients exhibited significant visuospatial neglect impairment on the Cancellation Task with 22 patients exhibiting both allocentric and egocentric neglect, 11 exhibiting significant egocentric neglect without allocentric neglect, and 2 patients presenting with only allocentric neglect with particularly severe allocentric scores of 10 and 18. All cases of egocentric and allocentric neglect in egocentric neglect dyslexia patients were found to impact the same spatial lateralization as these patients’ reading impairment.

Conversely, allocentric neglect dyslexia was found to be doubly dissociated from both egocentric and allocentric visuospatial neglect. 8 cases of right-lateralized allocentric neglect dyslexia were found to occur independently of any domain-general egocentric or allocentric neglect. There was no significant difference in the sentence reading percent correct between allocentric neglect dyslexia patients with and without neglect (F(1,15) = 2.077, p = .17). Of the 9 cases of allocentric neglect dyslexia which co-occurred with neglect, 2 cases (1 right and 1 left) were found to occur independently of egocentric neglect and 2 cases (both right lateralized) were found to occur in the absence of allocentric neglect. Interestingly, 2 cases involved right allocentric neglect dyslexia which occurred alongside left allocentric neglect. See Table 2 for a detailed breakdown per patient of the type and nature of the neglect dyslexia patient reading errors and the performance on the cancellation task, including ego- and allocentric asymmetries.

**Table 2.** Neglect dyslexia patient Reading Task and Cancellation Task performance along with basic stroke information. ICH = Intracerebral hemorrhage, TIA = Transient Ischemic Attack, NR = not reported in medical notes. ND = Neglect Dyslexia. On the cancellation task, Ego denotes the egocentric asymmetry, Allo denotes allocentric asymmetry. In both asymmetry scores positive scores reflect left-sided neglect, and negative scores reflect right-sided neglect impairments. Asterix represent significant impairment.

Patient Cognitive Assessment Performance										
	Reading Performance					Cancellation Task			Stroke	
	Neglect Dyslexia Reading Errors			Total	ND Side	Ego	Allo	Total	Type	Side
**P1**	thought – > “though”	colonel – > “colonial”		13/15	Right	0	0	40	Ischemic	Right
**P2**	thought – > “though”	colonel – > “colonial”	yacht – > “yach”	9/15	Right	−7*	−4*	39	Ischemic	Left
**P3**	any – > “an”	thought – > “though”	sitting – > “sit”	7/15	Right	−4*	0	45	Ischemic	Left
**P4**	thought – > “though”	colonel – > “color”		13/15	Right	−9*	2*	39	NR	Right
**P5**	colonel – > “nel”	yacht – > “cht”		2/15	Left	5*	4*	5	Ischemic	NR
**P6**	swords – > “words”	treasures – > “measures”		40/42	Left	9*	9*	9	ICH	Right
**P7**	belong – > “began”	castle – > “case”	winning – > “winner”	30/42	Right	−12*	0	32	Ischemic	Right
	a – > “at”	leisurely – > “leisure”	debating – > “depending”							
	jury – > “journey”	members – > “member”	impartial – > “impart”							
**P8**	belong – > “belongs”	we – > “we’ve”	concert – > concern	37/42	Right	4*	−4*	14	Ischemic	NR
	impartial – > “impartable”									
**P9**	islands – > “island”	thought – > “though”	colonel – > “cur”	10/15	Right	−1	0	49	NR	Left
	yacht – > “yach”									
**P10**	favorite – > “avorite”	trough -> “rough”		13/15	Left	−1	0	46	TIA	Left
**P11**	swords – > “sword”	award – > “aweer”	impartial – > “imperial”	34/42	Right	0	0	50	TIA	NR
**P12**	treasures – > “treasuries”	award – > “awards”	winning – > “winnings”	39/42	Right	1	0	46	TIA	NR
**P13**	swords – > “sword”	winning – > “winner”	treasures – > “treasure”	38/42	Right	0	0	50	ICH	Left
**P14**	swords – > “sword”	treasures – > “treasure”	members – > “member”	38/42	Right	1	0	48	Ischemic	Left
**P15**	treasures – > “treasure”	leisurely – > “leisure”		38/42	Right	0	−1	50	Ischemic	Left
**P16**	swords – > “sword”	belong – > “belong”	castle – > “cave”	30/42	Right	−3*	−1	36	NR	Left
	award – > “awardry”	winning – > “which”	debating – > “depending”							
	impartial – > “impartiate”									
**P17**	treasures – > “treasure”	members – > “member”		40/42	Right	0	3*	44	Ischemic	Right

**Figure 4.**
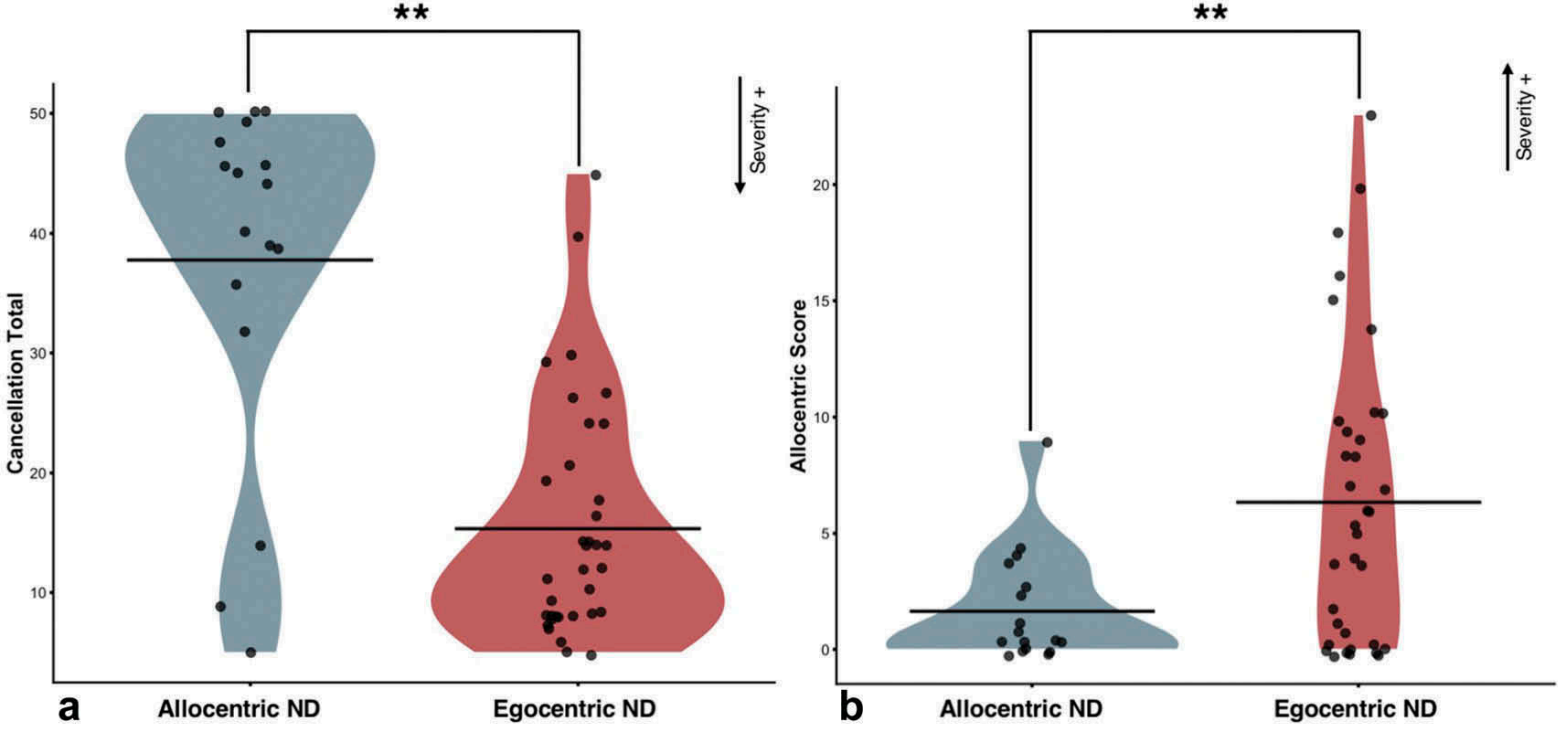
Differences in egocentric severity (Panel A) and allocentric severity (Panel B) between patients with neglect dyslexia and sentence level neglect. Low cancellation totals represent more severe egocentric impairment. High allocentric scores represent more severe allocentric neglect. ** = p < 0.01. Shaded areas illustrate the distribution of data points.

Next, a series of analyses were conducted to investigate the relationship between the severity of the visuospatial neglect within the subgroup of patients exhibiting neglect-related reading impairments. A mixed ANOVA, patients with egocentric neglect dyselxia were found to have significantly more severe egocentric (F(1,50) = 42.6, p = .002, η^2^ = .46) and allocentric (F(1,50) = 8.50, p = .005, η^2^ = .15) neglect than patients exhibiting allocentric neglect dyslexia (Figure 4).

A subset of 37 patients completed the OCS/BCoS Visual Field Task. 25/29 sentence-level neglect patients and 3/12 neglect dyslexia patients exhibited visual field deficits when measured this way, which impacted the same side of space as their reading impairment. Only 1 allocentric neglect dyslexia patient with no neglect (total N = 8) exhibited a visual field impairment.

## Discussion

Egocentric and allocentric neglect dyslexia were found to represent doubly dissociated, independent cognitive impairments. Egocentric neglect dyslexia was not found to occur independently of domain-general visuospatial neglect. Alternatively, allocentric neglect dyslexia was found to be doubly dissociated from both egocentric and allocentric visuospatial neglect impairment. The results of this study suggest that allocentric neglect dyslexia cannot be fully understood as a consequence of egocentric or allocentric visuospatial impairment. In contrast, we found no evidence for a dissociation between egocentric neglect dyslexia and visuospatial neglect.

This investigation demonstrated that egocentric and allocentric neglect dyselxia represent dissociated, independent cognitive deficits, replicating and extending previous studies which also reached this conclusion about dissociable processes (Beschin et al., 2014; Ptak et al., 2012). Importantly, this dissociation is frequently not accounted for when conducting research involving these reading impairments (Galletta, Campanelli, Maul, & Barrett, 2014; Leff & Behrmann, 2008). Failing to differentiate between these two independent cognitive impairments may potentially confound efforts to investigate these conditions’ underlying attentional biases, neural correlates, and recovery trajectories. We further extend previous research by investigating the role of object- or allocentric neglect in explaining word-centered reading impairments in allocentric neglect dyslexia.

We suggest that egocentric neglect dyslexia is best understood as a consequence of domain-general visuospatial impairment rather than an independent cognitive deficit. As in previous research (Beschin et al., 2014; Ptak et al., 2012), all cases of egocentric neglect dyslexia were found to co-occur with similarly lateralized visuospatial neglect. However, two egocentric neglect dyslexia cases occurring alongside severe allocentric but in the absence of domain-general egocentric neglect were identified in this investigation. There are several possible explanations for these cases of egocentric neglect dyselxia seemingly occurring in the absence of egocentric neglect. First, previous research has revealed that stimulus density modulates the severity of egocentric neglect impairment (Husain & Kennard, 1997; Kartsounis & Findley, 1994). It is therefore highly probable that egocentric neglect deficits may be exacerbated by the increased stimulus density and attentional demand associated with reading passages of prose compared to cancellation tasks (Riddoch, 1990). Second, it is possible that egocentric neglect dyslexia may be underpinned by more than one domain-general visuospatial impairment. For example, visual field impairments impacting one side of egocentric space are a frequent consequence of brain damage (Habekost & Starrfelt, 2006; Leff & Starrfelt, 2014). Patients with visual field impairments may struggle to scan passages of text and may therefore fail to report words presented on one side of space. Indeed, we found in 25 patients with egocentric neglect dyselxia, they also failed the confrontation task. Though this may indicate a co-occurring visual field impairment, it may also simply reflect the very severe neglect, where patients fail to identify any contralesional stimuli. Though the OCS can pinpoint a visual field deficit without neglect (i.e., unimpaired cancellation task), when both tasks are impaired, the two problems cannot be teased apart without further measures.

We suggest that while egocentric neglect may not be the only possible cause of egocentric neglect dyselxia impairment, this condition is best understood as a consequence of domain-general visuospatial impairment rather than a content-specific reading deficit. In contrast, allocentric neglect dyslexia was found to be dissociated from both egocentric and allocentric visuospatial neglect. This investigation identified two patients who exhibited right allocentric neglect dyslexia which co-occurred with left allocentric neglect, illustrating that this dissociation applies not only to the occurrence of these conditions but also to their lateralization. These findings align with previous research suggesting that some cases of allocentric neglect dyslexia represent content-specific impairments which are not caused by domain-general visuospatial deficits (Caramazza & Hillis, 1990; Friedmann & Nachman-Katz, 2004; Moore & Demeyere, 2018). However, as some cases of allocentric neglect dyslexia were found to co-occur with domain-general neglect it seems that this explanation cannot be applied to all cases of this impairment.

Our findings are in line with a conceptualization of neglect dyslexia as a heterogenous condition with some cases being attributable to domain-general impairments and other cases to content-specific cognitive problems (Hillis & Caramazza, 1995; Moore & Demeyere, 2017). Our data does not support a view that allocentric neglect dyslexia, in the absence of visuospatial neglect can be fully explained by other domain-general vision impairments (e.g., hemianopia, quadrantanopia) (Habekost & Starrfelt, 2006). Only 1 patient with allocentric neglect dyslexia in the absence of visuospatial neglect exhibited significant visual field impairment on the confrontation test. Additionally, visual field impairments can often be compensated for by visual scanning of written stimuli meaning that it seems unlikely that visual field impairment could explain the remaining occurrences of allocentric neglect dyslexia. Similarly, previous authors have argued that allocentric neglect dyslexia which appears to occur independently of domain-general neglect may be attributed to subtle neglect deficits which are exacerbated by the increased stimulus density and attentional demand associated with reading arrays of text (Jackson & Coltheart, 2013; Riddoch, 1990; Riddoch, Humphreys, Cleton, & Fery, 1990). However, this explanation cannot account for patients who exhibit allocentric neglect dyslexia and domain-general neglect in conflicting lateralizations and therefore, at best, can only explain a subset of allocentric neglect dyslexia cases.

Previous research has provided a framework for interpreting allocentric neglect dyslexia as a content-specific impairment. Some cases of allocentric neglect dyslexia may be best understood as an impairment occurring at a graphemic-level where words’ spatial information is encoded at a representational level within the orthographic lexicon (Caramazza & Hillis, 1990; Hillis & Caramazza, 1995; Jackson & Coltheart, 2013). Hillis and Caramazza (1995) propose a three-tier model of neglect dyslexia in which reading errors can occur within a retinocentric (egocentric), object-centered, or word-centered frame of reference. This model seems to neatly account for differential performance of allocentric neglect dyslexia as well as providing a theoretical framework which can help explain why some patterns of allocentric neglect dyslexia might not be related to domain-general spatial biases. Impairment to domain-general visual processes might produce a retinocentric neglect dyslexia error pattern but impairment to a graphemic level of spatial representation could plausibly result in a content-specific impairment.

Allocentric neglect dyslexia likely represents a highly heterogeneous condition with some cases being caused by domain-general impairments, content-specific impairments, or a combination of the two (Hillis & Caramazza, 1995; Moore & Demeyere, 2017; Vallar et al., 2010; Young et al., 1991). Future investigations need to account for the complexity of this syndrome if understanding of allocentric neglect dyslexia’s underlying cognitive impairments is to be advanced.

The implications of this investigation are particularly relevant when considered in the context of current post-stroke rehabilitation approaches. If egocentric neglect dyslexia reading impairment is caused by domain-general visuospatial deficits, this reading impairment would be expected to benefit from rehabilitation strategies which improve these domain-general impairments. Many egocentric neglect rehabilitation strategies have been proposed (Bowen & Lincoln, 2007; Bowen & Wenman, 2002; Yang, Zhou, Chung, Li-Tsang, & Fong, 2013) as well as several specifically designed to ameliorate reading impairment in patients with domain general visuospatial impairment (Leff & Behrmann, 2008). However, although many cases of allocentric neglect dyslexia appear to be a result of these domain-general attentional biases it seems likely that not all patients with this reading impairment will benefit from domain-general rehabilitation. It is therefore critically important for future research to design and develop allocentric neglect dyslexia specific rehabilitation strategies which aim to help patients suffering from this reading-specific impairment in the absence of a domain-general neglect impairment.

### Limitations

Several potential limitations were encountered in this investigation. First, the OCS and BCoS are intended to serve as multidomain screening tools rather than detailed cognitive assessments. For this reason, these screens’ reading tasks are comparatively very short with each patient reading between 15–42 words. These brief reading tasks, combined with the stringent criteria employed for classification, were likely only sensitive enough to detect more severe cases of neglect-related reading impairment. Similarly, these reading tasks were designed to screen for and detect several forms of acquired dyslexia and are therefore not specifically optimized for detecting neglect dyslexia. The current findings therefore cannot be employed to accurately calculate the incidence rate of neglect-related reading impairments. This limitation however, does not influence the accuracy of the conclusions regarding neglect dyslexia error patterns within the patients who were identified. Additionally, previous research has strongly suggested that administering multiple, separate tests of neglect is the most reliable method for identifying visuospatial neglect impairment in patients (Gottesman et al., 2010; Halligan, Cockburn, & Wilson, 1991; Huygelier, Moore, Demeyere, & Gillebert, In Review; Lindell et al., 2007). However, as this investigation employed existing data it was not possible to include additional neglect tests.

Of the 17 patients exhibiting clear allocentric neglect dyslexia in this study, clinical admission CT scans were available only for 11 patients, and 4 of these scans showed no visible lesions. This is not unexpected as admission CT scans do frequently produce false negatives due to the time it takes for cerebrospinal fluid to occupy the lesioned space (González, 2005; Merino & Warach, 2010). The remaining 7 patients with lesion data presented too small a subset to produce sufficiently powered results, meaning that this study was not able to investigate the neural correlates of neglect dyslexia. Although previous investigations have identified cases of allocentric neglect dyslexia occurring in the absence of contralateral lesions (Friedmann & Nachman-Katz, 2004; Moore & Demeyere, 2018), there is a chance that cases of ipsilesional allocentric neglect dyslexia or reading impairment following a TIA were lasting consequences of a previous cerebrovascular accident which was not recorded in patients’ most recent medical notes. For this reason, this study does not attempt to draw conclusions relating to stroke type or side and neglect dyslexia impairment.

### Conclusions

The findings of this investigation clearly demonstrate that allocentric neglect dyslexia cannot be fully explained as a consequence of allocentric visuospatial neglect. Similarly, this study suggests that neglect-related reading impairments may be best understood as a cluster of dissociable reading impairments rather than a unitary syndrome. It is therefore critically important for future research to elucidate the specific cognitive mechanisms underlying neglect-related reading impairment to account for this heterogeneity and for new and tailored rehabilitation strategies to be designed and tested.
